# “Hadrontherapy for Life” Symposium, Caen, March 10/11, 2025–Strategy for the Future–Pancreatic Cancer

**DOI:** 10.1016/j.ijpt.2025.101287

**Published:** 2025-11-26

**Authors:** Jean-Louis Habrand, Siamak Haghdoost, Semi B. Harrabi, Cindy Neuzillet, Véronique Vendrely, Ellie Light, Jean Lubrano, Tatsuya Ohno, Shigeru Yamada, Zheng Wang, Jerôme Doyen, Remi Dendale, Dinu Stefan, Anita Mahajan, Bradford Hoppe, Arnold Pompos, Juliette Thariat

**Affiliations:** a Radiation Oncology Department, François Baclesse Cancer Center, Caen, France; b ABTE/ToxEMAC Laboratory, University of Caen Normandy, Caen, France; c Radiation Oncology Department, Heidelberg University Hospital, Heidelberg, Germany; d Digestive Medical Oncology Department, Institut Curie, Paris, France; e Radiotherapy Department, Haut-Lévêque University Hospital, Bordeaux, France; f Radiotherapy Department, The Christie NHS Foundation Trust, Manchester, United Kingdom; g Digestive Surgery Department, University Hospital Caen, Caen, France; h Department of Radiation Oncology, Gunma University Graduate School of Medicine, Gunma, Japan; i QST Hospital, National Institutes of Quantum and Radiological Science and Technology, Chiba, Japan; j Department of Radiation Oncology, Shanghai Proton and Heavy Ion Center, Shanghai, China; k Radiation Oncology Department, Antoine Lacassagne Cancer Center, Nice, France; l Radiation Oncology Department, Institut Curie, Paris, France; m Radiation Oncology Department, Mayo Clinic Rochester, Rochester, MN, USA; n Radiation Oncology Department, Mayo Clinic Florida, Jacksonville, FL, USA; ° Radiation Oncology Department, University of Texas Southwestern Medical Center, Dallas, TX, USA

**Keywords:** Pancreatic cancer, PDAC, Radiotherapy, Hadrontherapy, Carbon therapy

## Abstract

The symposium “Hadrontherapy for Life,” held in Caen, on February 10 and 11, 2025, brought together over 100 international experts of heavy ions particle therapy. Clinical aspects of current indications and future strategies were discussed. Pancreatic cancer that may reach the second cause of cancer mortality in the next decade, was prioritized. Stimulating clinical data accumulated in Japan and more recently in Europe suggest an important role for carbon ion radiotherapy (CIRT) in advanced presentations but also in a preoperative setting, at the price of acceptable toxicity. Biological aspects also plead for combinations of CIRT with bio or immune therapy.

## Introduction

The symposium “Hadrontherapy for Life” included discussions by experts on potential clinical indications that could be strategic for future heavy ion radiotherapy (HIRT) programs like those at the CYCLHAD particle therapy center in Caen. Accepted worldwide indications are reported in a separate paper of this issue. For the “strategy session,” we selected two topics for in-depth evaluation in the context of modern strategies and technologies: pancreatic adenocarcinoma and pediatric tumors. The former detailed in this article is still familiar to carbon ion radiotherapy (CIRT) experts, although its management remains highly challenging and requires improvement.

## Presentation and work-up

Rare in the past, the incidence of pancreatic carcinoma has increased considerably since the 1980s: 60 000 in US, 146 000 in Europe, and 16 000 cases in France in recent statistics (Global Cancer Observatory data, https://gco.iarc.who.int.). With over 138 000 fatal cases in Europe per year, 49 000 in US, 14 500 in France, it is currently the third to fourth leading cause of oncological mortality and is projected to rise to the second leading cause by the decade spanning 2030 to 2040. The association with tobacco, diabetes mellitus (type 2), obesity, chronic pancreatitis (including alcohol-related), genetic syndromes (Peutz-Jeghers, Lynch, BRCA1 and 2, FAMMM) and family history of cancer and of precancerous cystic lesions (IPMN and MCN) are advocated.^1^ Some germ line mutations look also over-represented (including BRCA1 and 2, PRSS1, p16/CDKN2A).^2^ Its diagnosis remains late due to the unspecific clinical presentation with lack of warning symptoms or misleading ones (“back pains” …), with consequently a large proportion of inoperable and metastatic stage at presentation. Initial work-up is crucial to determine the patient’s performance and nutritional status (ie, tolerance to treatment), and tumor extension (local, nodal regional, or distant) by enhanced thin slice CT and MRI. FDG- or FAPI-PET may also be helpful for tumor evaluation before and after induction chemotherapy (CT) This staging should clarify extension and resectability status (resectable, border-line resectable, locally advanced, or metastatic) that conditions further strategy.^1^

## Management

1. Surgery (pancreato-duodenectomy or left pancreatectomy, according to primary tumor location), in combination with CT, remains the only potentially curative one. This can only be achieved in 10%-15% cases upfront resectable. Pathologically, invasive pancreatic ductal adenocarcinoma (PDAC) is the most common finding.^3^ Among borderline or locally advanced patients (ie, based on invasion of main vessels), the administration of initial “induction” CT ± radiation therapy (RT) makes additional patients eligible for surgery: 60% to 70% of the former and 20% to 30% of the latter. The operative mortality varies from 5% to 10% and serious or even lethal complications (mainly pancreatic fistula) affecting 1 of 2 patients. Only a microscopically complete excision (R0) allows prolonged survival, a rare event as evidenced by the median survival below 2 years and a 5-year survival of 13%.^4^ Minimally invasive surgery performed laparoscopically or robotically does not appear to affect prognosis but positively wound complications and impact on immune function (after left pancreatectomy).
2. Pre- and/or post-operative CT, a long disappointment (administration of gemcitabine alone) has enjoyed a renowned interest with new regimens: FOLFIRINOX (oxaliplatin, irinotecan, 5-Fluorouracil, folic acid), S1 (Tegafur, Gimeracil, Oteracil), PAXG (Capecitabin, Cisplatin, nab-Paclitaxel, gemcitabin)…^2^, ^5^ which can be potentiated further by an electric field belt,^6^ especially in borderline resectable presentations. Recent advances have also concerned molecular biology in terms of prognosticators (BRCA1 and 2, SMAD4, TP53), targetable by biotherapy (PARP inhibitors) or immunomodulation (vaccine in adjuvant setting, CAR T cells, and CD40 agonists) especially in metastatic presentations.^7^ Many studies are ongoing.
3. “Conventional” x-ray RT, a historical but highly controversial adjunct to surgery, faces many difficulties: radio-resistant tumor populations requiring high doses (50 Gy, fractionated, or even more in case of residual macroscopic disease), in contrast with the lower tolerance of the numerous organs at risks in the vicinity (small bowel, liver, kidneys, spinal cord).^8^ It is not surprising if it is not even part of some recent recommendations of international cooperative groups like European Society of Medical Oncology (ESMO)^9^, ^10^ although it remained integrated into past and recent chemo-radiotherapy research programs: LAPC07 (patients in R1 resection), PREOPANC3, ESPAC 5, PANDAS-PRODIGE 44, CONKO-007 (preoperative regimens).^11^, ^12^, ^13^
4. Highly targeted RT (allowing for improved organs at risks sparing and/or dose escalation) raised high hope and an actual trend for improvement in the local outcome dealing with x-rays intensity modulation,^14^ motion-control,^15^ MRI-guided,^16^ stereotactically multitargeted,^17^, ^18^, ^19^, ^20^, ^21^ or protontherapy (PT)^22^, ^23^, ^24^ (Table 1).

**Table 1:** Radiotherapy in advanced and inoperable pancreatic carcinomas (for typographic reasons, figures are approximated).

Author (Date) *Journal [Ref]*	Stage	#Pts *FUp*	RT	CT	Outcome	Toxicity *(RT related)*	Comments
x-rays RT							
Pollom et al (2014) *Int J Radiat Oncol Biol Phys* ^18^	Inop M0	167 *8m*	30 Gy SBRT (1-5f)	GEM or Folfirinox	1YOS: 31%-35% 1YLC:88%-90%	G3 + 4:12%	RT hypofrac BED_T_: 55-87 Toxicity 1f>5f
Prasad et al (2016) *Pract Radiat Oncol* ^14^	Inop, M0	205 *22m*	3D vs IMXRT. 50 vs 56 Gy	GEM or Folfox	Med 0S: 15 m OS 3D = IMXRT	3D:34% IMXRT:16%	Not randomized
Hassanzadeh et al (2021) *Adv Radiat Oncol* ^20^	Inop, M0	44 *16m*	SBRT, MRI-G 50 Gy (5f)	-	2Y OS:38%	G2:7% G3: 5%	RT hypofrac BED_T_ 98-100
Reyngold et al (2025) *JAMA Oncol* ^21^	Inop*)* M0	25 *39m*	IMXRT, MRIG 50-75 Gy (5-25f)	GEM or Folfirinox	Med OS: 22 m 2Y OS: 44%	G3:12%	Inop for medical CI
PT							
Terashima et al (2012) Radiother Oncol ^22^	Inop M0	50 *12.5m*	50-67.5 GyEq (25-26f)	GEM	1YOS: 77% 1YLC: 82%	G3: 10%	RT hypofrac
Hiroshima et al (2019) Radiother Oncol ^23^	Inop M0	42 *14m*	PT±hyperthermia 50-67.5 GyEq	GEM or S1	2YOS: 51% 2YLC: 79%	G3: 40% G4: 5%	
Ogura et al (2022) Radiat Oncol ^24^	Inop M0	123 *15m*	67.5 GyEq	GEM	2YOS:36% 2YLC:59%	G3:42% G4:2%	
CIRT							
Shinoto et al (2016) *Int J Radiat Oncol Biol Phys* ^30^	Inop M0	76 >24m	43-55 GyEq	GEM	2YOS:35% (55 Gy = 48%) 2YLC:83%	G3:1%	RT Hypofrac Includes St IV (N+)
Kawashiro et al (2018) *Int J Radiat Oncol Biol Phys* ^31^	Inop M0	72 *14m*	53-55 GyEq (12f)	GEM Or S1	2YOS:46% 2YLC: 76%	G3: <5%	RT Hypofrac
Shinoto et al (2018) *Radiat Oncol* ^32^	Inop M0	64 *24.5m*	55GyEq	GEM or S-1	2YOS:53% 2YLC: 82%	G3:3%	RT Hypofrac
Liermann et al (2022) *Strahlenther Onkol* ^33^	Local Relapse	13 9.5m	48GyEq (12f)	-	2YOS: 25% *(10 DC: Mets)*	G3:8%	Poor Met control

**Abbreviations: DC, dead; f, fraction; hypofrac, hypofractionated; G, grade; GEM, gemcitabin; GyEq, Gray RBE Equivalent; Inop, inoperable; LC, local control; m, month; Med, median; MRIG, MRI-Guided; Met, metastases; OS, overall survival; St, stage; Y, year; Other abbreviations: see text**.

## Contribution of heavy ions radiobiology

A growing body of evidence helps understand the biology of PDAC radio-resistance. It implicates cancer stem cells (CSCs), the tumor’s microenvironment, antioxidant defenses, and DNA repair capacity.

1. According to the CSC hypothesis, only a small subset of tumor cells possesses the ability to sustain long-term self-renewal, drive differentiation, and initiate new tumor growth. Importantly, CSCs exhibit phenotypic plasticity: they may originate not only from pre-existing stem-like tumor cells (CD44+/CD24+) but also through the de-differentiation of more differentiated tumor cells, particularly in response to radiation stress.^25^
2. The stroma (fibroblasts, extracellular matrix, and immunesuppressive cells) limits vascular perfusion, creating regions of severe hypoxia, and activates transcriptional programs that further enhance radioresistance. Hypoxia-inducible factors (HIF-1α and HIF-2α) drive the expression of genes involved in angiogenesis (eg, VEGF), glucose metabolism (glycolytic enzymes), epithelial–mesenchymal transition, and stemness-associated transcription factors.^26^
3. Reactive oxygen species (ROS) damage DNA, proteins, and lipids. CSCs possess potent antioxidant systems that neutralize ROS and reduce radiation lethality. The master regulator of this response is the transcription factor Nrf2 (nuclear factor erythroid 2-related factor 2).^27^ PDAC CSCs are characterized by elevated Nrf2 activity, which confers a survival advantage in the face of radiation-induced ROS.
4. DNA damages and repair mechanisms (DDRs) of CSCs manifest through activation of DDR proteins such as ATM, ATR, DNA-PK, and Rad51, enabling efficient repair through homologous and non-homologous recombination.^28^

## Contribution of heavy-ions radio-physics

Ballistic advantages of the Bragg curve are well known in terms of high-dose delivery to tumors and reduced exposure of surrounding normal tissues. A better understanding of heavy ions interactions with matter (ie, scattering and nuclear interactions) along the path and beyond the fall-off of physical dose (the so-called “radioactive tail”) could contribute to a better understanding of heavy ions specificities, such as: 1/ variable LET/RBE models from entrance-beam to range-end for different particles that sustain optimized treatment planning; 2/ carcinogenic aspects.^29^

## Contribution of heavy ions clinics

In Japanese series, CIRT produces results clearly superior to those published by other modern RT technologies in advanced forms of very poor prognosis: ≥50% median survival at 2 years vs 30%-40% (including PT)^30^, ^31^, ^32^, ^33^ (Table 1). The only 5-year survivors are in the CIRT group. The experience of patients living without disease for 8 years was presented on site (Yamada S, personal communication). A Chinese pilot study reports on a local control of 88% at 1 year and 73% at 2 years (Wang Z, unpublished data). Its program included respiratory synchronization during the session and an on-line check by in vivo dosimetry (PET scan).

Noteworthy CIRT has also been tested in Japanese studies in a preoperative setting with highly promising and durable outcome: 52% at 5 years (42% including secondary contra indication for surgery) in Shinoto’s et al series^34^ along with acceptable toxicity: G3 + 4 = 8%. Fascinating observations relate to the potential impact of CIRT on tumor environment, and metastatic sites (bystander, cohort and abscopal effects).^35^ These intriguing phenomena suggest immunological implications, which are comforted by the negative impact on survival of persistent post treatment lymphopenia observed after x-rays therapy but not CIRT.^36^

## Strategy for the future

1. Harmonizing approaches for CIRT treatment planning in PDAC to ensure reproducible results at different centers and to enable multi-institutional and multi-continental clinical trials.^37^, ^38^
2. Improving local control by escalating the biological dose of heavy ions to tumor volume. Exploring different time- and dose-fractionation schedules according to the European and Japanese approaches. Designing further intercomparisons between low and high LET radiation in a randomized fashion.
3. Evaluating short- and long-term toxicities of heavy ions. Exploring the benefit of “all heavy ions” vs combined low (ie, x-rays or protons) and high LET radiations.
4. Improving distant control of subclinical metastases based on recent innovative therapies (biologic agents, immunotherapy, and vectorized internal radiotherapy) and prognostic markers (see above).
5. Improving distant control of overt clinical metastases based on new chemotherapeutic regimens and innovative therapies.
6. Ancillary studies:
  • Understanding biological mechanisms that sustain radioresistance of PDAC and develop new models to overcome it.
  • Exploring bystander and abscopal effects through case reports and biological investigations
  • Evaluating HIRT oncological outcome and toxicity according to competing biological/dosimetrical models (eg, LEM vs MKM)^39^, ^40^

## Discussion

Many data converge towards the use of CIRT in pancreatic tumors, where “conventional” RT holds a minimal place. The increasing incidence and poor prognosis of this disease strongly encourage the exploration of innovative treatments.

Radiobiological data point out radioresistance as a multifactorial key-factor involving CSC biology, hypoxia-driven signaling, antioxidant defenses, and DNA repair capacities^41^; the importance of inhibition of HIF signaling (either by genetic tools or pharmacological agents); inhibition of Nrf2, still explored in Glioblastoma cell lines,^42^ targeting DDR pathways (PARP, ATM, ATR, and DNA-PK inhibitors). Hyperthermia has also been long tested since tumor cells are sensitive to heat. A model was recently developed based on paramagnetic nanoparticles implanted in situ and then exposed to an alternating magnetic field.^43^

Clinically, CIRT seems attractive in inoperable forms (locally advanced), but also in operable ones. A recent evaluation by Yamamoto et al concluded that local control was close by the conventional approach (surgery + CT) or CIRT alone.^44^ Although survival deteriorated in the second group beyond 2 years, this was a highly inspiring message for future associations of CIRT + CT that could improve the metastatic outcome and so survival. Other options could be the association of CIRT with targeted or immunomodulatory drugs, radio-immunotherapy, nanoparticles….

A recent meta-analysis^45^ of CIRT indications, that classified evidence as “strong,” “conflicting,” or “weak,” ranked PDAC in the second group due to the lack of phase III studies. Until now most groups have considered phases I or small phases II only. More ambitious programs should consider a large patients’ accrual at the price of increased team workload and adequate resources. Multicentric and international studies have emerged recently. PIOPPIO trial in a pre-operative setting: 3 cycles of neoadjuvant FOLFIRINOX followed by a short course CIRT for resectable or borderline resectable PDAC (CNAO, Italy), phase III CIPHER trial (US-Japan), and phase II PACK study (HIT, Germany).^46^ Most results are pending.

## Conclusion

The symposium clearly showed the clinical interest of high LET particles in addition to, or in combination with, low LET protons to combine ballistic selectivity (to better spare normal tissues from deleterious effects of radiation) and original biological properties (great interest in selected highly radio-resistant processes). Multi-ions combinations or flash dose-rate (ie, >100 Gy/sec) have also been proposed.^29^ CNAO published at the time of the symposium an elegant and inspiring proposal that mixes clinical and biological considerations to select patients in a long run^47^: as far as biology guidelines, it stresses altering the basic concept of the interaction of ionizing radiation with biological tissues known as the “4Rs” (“Repair, Reoxygenation, Repopulation, Redistribution in the cell cycle)” with two extras: “intrinsic Radiosensitivity,” and “immune Reactivation,” 2 concepts of particular interest in HIRT research programs. As far as clinical guidelines, the Italian group also proposes the “4Rs” dedicated to CIRT indications: “rare,” “radio-resistant,” “re-irradiation,” “radio-induced,” with multiple “Rs” combinations in the same patient being prioritized. Pancreatic cancer is becoming a health priority and justifies certainly major initiatives like the impact of heavy ions in the modern armamentarium at a national and international level.

## Declaration of Conflicts of Interest

The authors have no conflicts of interest to disclose.
